# Supplementary material for: Microbial assemblages and methanogenesis pathways impact methane production and foaming in manure deep-pit storages
Source: PLoS One. 2021 Aug 3;16(8):e0254730. doi: 10.1371/journal.pone.0254730 (PMC8330953; doi:10.1371/journal.pone.0254730)
Supplement: S1 Table — (PDF) [file pone.0254730.s005.pdf]

Integrator[a] ID	Feedmill ID	# of Pumping Events[b]	# of Farms
1	1	1	3
		2	16
		3	11
		4	1
	2	1	2
		2	5
2	3	1	1
		2	15
		3	3
		4	1
	4	1	1
		2	8

a. Company that provides pigs, feed, and other services to a contract grower. Integrators provides consistent genetics, feed, and management.

b. Number of times manure was removed from the storage pit.
