# Supplementary material for: Microbial assemblages and methanogenesis pathways impact methane production and foaming in manure deep-pit storages
Source: PLoS One. 2021 Aug 3;16(8):e0254730. doi: 10.1371/journal.pone.0254730 (PMC8330953; doi:10.1371/journal.pone.0254730)
Supplement: S2 Table — The final average bacterial sequencing depth and coverage were reported in columns 3 and 4. The final average methanogen sequencing depth and coverage were reported in columns 6 and 7. (PDF) [file pone.0254730.s006.pdf]

Manure Surface Texture	Bacteria			Methanogens		
		Average			Average	
	Number of Samples	Sequencing Depth	Good's Estimate of Coverage	Number of Samples	Sequencing Depth	Good's Estimate of Coverage
No-foam	162	35328	0.9902	10	9885	0.9964
Crust	98	28436	0.9905	14	9288	0.9948
Foam	228	25786	0.9913	19	8494	0.9981
