# Supplementary material for: Microbial assemblages and methanogenesis pathways impact methane production and foaming in manure deep-pit storages
Source: PLoS One. 2021 Aug 3;16(8):e0254730. doi: 10.1371/journal.pone.0254730 (PMC8330953; doi:10.1371/journal.pone.0254730)
Supplement: S3 Table — Bray-Curtis dissimilarities were calculated using operational taxonomic unit (OTU) relative abundance. (PDF) [file pone.0254730.s007.pdf]

Factor	Bacteria[e]			Methanogens [e]		
	R^2	F	P	R^2	F	P
Farm	0.48	8.93	1	0.83	4.42	1
Feed Mill	0.15	29.04	1	0.26	4.61	0.001
Surface Texture[a]	0.12	32.31	0.001	0.3	8.67	0.239
Foaming Status[b]	0.1	52.84	0.001	0.26	14.37	0.001
Date[c]	0.08	3.4	0.001	0.08	1.76	0.005
Integrator[d]	0.07	36.03	1	0.08	3.71	0.001

a. Non-foaming samples (No-foam), crust forming samples (Crust), and foaming samples (Foam)

b. Samples observed with foams (Foam) and samples that were absent of foams (No-foam and Crust)

c. By month and year

d. Company that provides pigs, feed, and other services to a contract grower. Integrators provides consis

e. Sampling farms as experimental blocks
