# Supplementary material for: Microbial assemblages and methanogenesis pathways impact methane production and foaming in manure deep-pit storages
Source: PLoS One. 2021 Aug 3;16(8):e0254730. doi: 10.1371/journal.pone.0254730 (PMC8330953; doi:10.1371/journal.pone.0254730)
Supplement: S1 File — (DOCX) [file pone.0254730.s008.docx]

# **The correlations of the core microbial community in manure with different surface texture**

Permutational multivariate analysis of variance revealed that individual farms explained the largest variations observed in the bacterial (S2 Table) and methanogen communities. To minimize the community variations unique to individual farms, microbial OTUs present in all samples associated with the same surface texture were selected to represent core no-foam, crust, and foam bacteria communities (e.g., “core” no-foam, crust, and foam communities).

The relative abundance of core bacteria identified at the genus level and core methanogen identified at the species level in no-foam, crust, and foaming samples were compared. The alternative hypotheses of how the relative abundance of a microbial genus differed among three types of manure were tested against the null hypothesis (H_0_: no-foam = crust = foaming) and evaluated using Bayes factors (B_10_).

The potential interactions between the core OTUs (raw abundance counts) and dietary inputs or manure characteristics were evaluated using Spearman’s correlation coefficients and Hoeffding dependence coefficients. Based on the coefficient distribution of significant relationships, we defined the top 5% of the correlation/dependence coefficient as strong potential interactions (greater or equal to 0.75 as a strong interactions related to methanogens, 0.44 for all bacteria-physicochemical parameter interactions in no-foam samples, 0.42 for all bacteria-physicochemical parameter interactions in crust samples, and 0.46 for all bacteria-physicochemical parameter interactions in foaming samples). To present the most prominent correlations, OTUs belonging to the same class and sharing the same significant correlations were grouped to show correlation at class level by averaging the correlation coefficients. The correlation analysis was performed in R (3.2.4) ^1^ using packages Hmisc (3.17-3).

# **Stability of the microbial communities**

Community stability over time is associated with underlying community structure dynamics. In manure, we expected that bacterial communities would become increasingly different from each other as time progressed. The relationship between time and community dissimilarity has been described in a model by ^2^:

where “a” represents the community dissimilarity initially (at time 0), “b” estimates the stability of the community, and “t” is the time elapsed. To evaluate the manure community stability, we calculated the bacterial community dissimilarities (Bray-Curtis distance) over time for samples taken from the same barn. Non-linear least square regression was used to estimate parameters “a” and “b.” If at two given time points (e.g., October, 2012 and December, 2012), a community shifted from one surface texture to another (e.g., no foam to crust), this community dissimilarity value for the given time elapsed (e.g., 2 months) was excluded. The Mantel test was used to estimate how well the time describes the community changes. For each surface texture category, dissimilarity calculations were bootstrapped (999 replicates using R package boot 1.3-18) to estimate the distribution of estimated parameter “b.” Two communities associated with different surface texture were considered to have significantly different in stability when the overlapped region of the estimated “b” distribution was less than 5%. This analysis could not be performed on the methanogen communities because of the small sample size.

**The statistical inference of Bayes factor**

A Bayes factor (BF_10_) describes the ratio of the Bayesian probability of the alternative hypothesis occurring over Bayesian probability of the null hypothesis ^3^. This can be shown in equation:


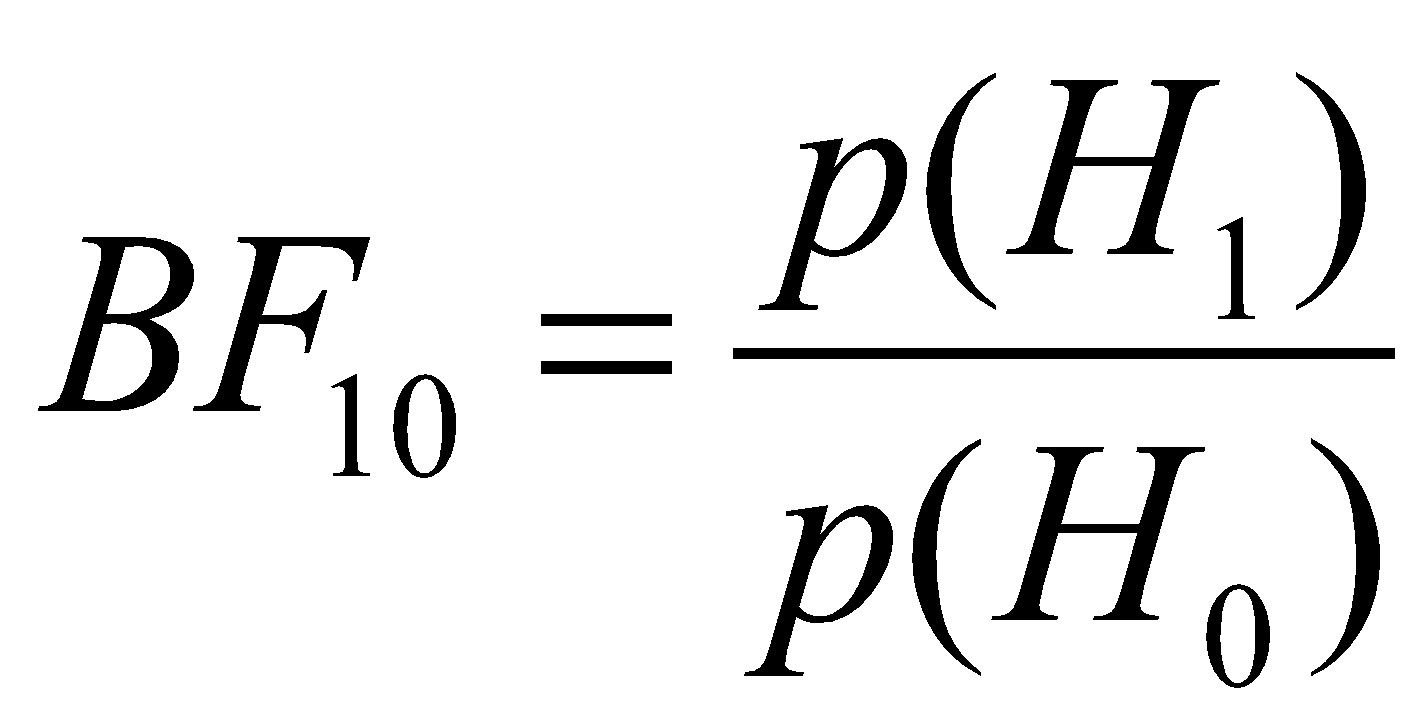


where p(H_1_) represents the probability of alternative hypothesis occurring, and p(H_0_) represents the probability of null hypothesis occurring. A BF_10_ greater than 20 was indicative of strong evidence to reject the null hypothesis. The odds of alternative hypothesis occurring in given data (i.e., posterior probability) can be calculated as:


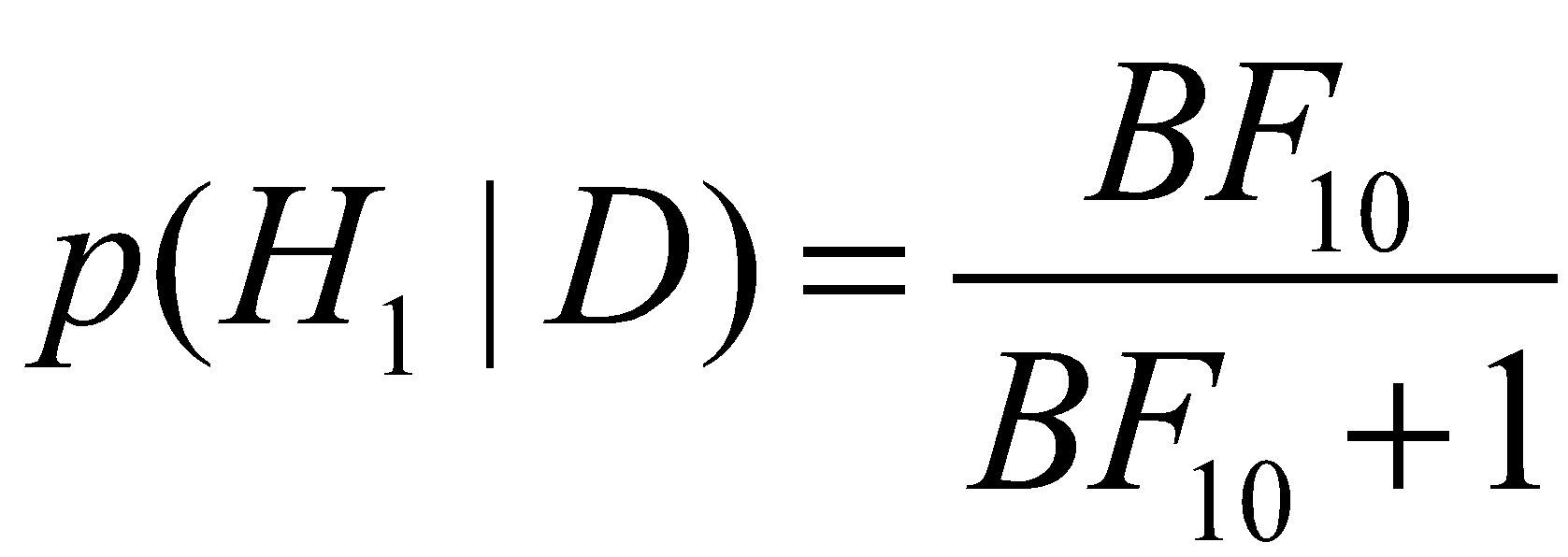


where D represents the data. Therefore, for BF_10_ ≥ 20, the likelihood for alternative hypothesis occurring is greater or equal to 0.953 and the likelihood for null hypothesis, p(H_0_|D), occurring is less than 0.05. Hence, we report hypothesis tests with a BF_10_ ≥ 20 as statistically significant in the paper.

**References**

(1) R Core Team. R: A Language and Environment for Statistical Computing. Vienna, Austria 2012.

(2) Baselga, A. Separating the two components of abundance-based dissimilarity: Balanced changes in abundance vs. abundance gradients. *Methods Ecol. Evol.* **2013**, *4* (6), 552–557.

(3) Kass, R. E.; Raftery, A. E. Bayes Factors. *J. Am. Stat. Assoc.* **1995**, *90* (430), 773–795.
