# Supplementary material for: Microbial assemblages and methanogenesis pathways impact methane production and foaming in manure deep-pit storages
Source: PLoS One. 2021 Aug 3;16(8):e0254730. doi: 10.1371/journal.pone.0254730 (PMC8330953; doi:10.1371/journal.pone.0254730)
Supplement: Attachment — Submitted filename: plosone_response_to_reviewers_2.docx [file pone.0254730.s010.docx]

Reviewer #1: The authors have adequately addressed the reviewers comments. One additional question arises:
L386: This does not make sense to the reviewer. Hydrogenotrophic methanogens oxidize H2, and use the reducing equivalents to reduce CO2. How do they oxidize acetate?

**Response:** We thank the reviewer for the question. Acetate oxidation in the absence of acetoclastic methanogens is part of the overall anaerobic methane production pathway. However, acetate oxidation is carried out by microorganisms other than hydrogenotrophic methanogens. We rephrased the sentence to clear the confusion.

Minor edits:

**Response:** We thank the reviewer for the suggestions. The edits below have been incorporated.

Title: Suggest deleting “on”.
L43 and L62: Please use SI units (meters, rather than ft).
L119, L131: Change “less” to “fewer”.
L256: Change “microorganism metabolism” to “microbial metabolism”.
L257: Change “many” to “most”.
L279-281: This is a little confusing as written. Suggest changing “Under standard anaerobic conditions, the breakdown of LCFA is carried out via acetogenesis [35,42]. This process is energy-consuming and non-spontaneous.”, to “Under anaerobic conditions, the breakdown of LCFA is carried out via acetogenesis [35, 42]. This process is endergonic and does not occur spontaneously under standard conditions.”
L298: Insert “, respectively” after “methanogens”.
L458: Change “fibers” to “fiber”.

Reviewer #2: Authors revised manuscript, and revised version of manuscripts responded answers to comments line by line, and issues raised have been resolved.

Reviewer #3: (No Response)
